# A Cortical Stress Signature of Fear‐Induced Walking: Mobile Electroencephalography Theta Suppression in Older Women With Fear of Falling

**DOI:** 10.1111/psyg.70219

**Published:** 2026-10-08

**Authors:** Guilherme Augusto Santos Bueno, Flávia Martins Gervásio, Anabela Correia Martins, Ruth Losada de Menezes

**Affiliations:** ^1^ Department of Medicine ‐ Center for Innovation and Research in Clinical Neuroscience University of Rio Verde (UniRV) Rio Verde Brazil; ^2^ Department of Medicine Euro‐American University Center (UNIEURO) Brasília Brazil; ^3^ Department of Physiotherapy State University of Goiás (UEG) Goiânia Brazil; ^4^ Coimbra Health School Polytechnic University of Coimbra Coimbra Portugal; ^5^ Center for Rehabilitation Research, Health School, Polytechnic of Porto Porto Portugal; ^6^ SUScita—Research Group on Sustainability, Cities and Urban Intelligence Coimbra Portugal; ^7^ Interdisciplinary Center on Aging Federal University of Goiás (UFG) Goiânia Brazil

**Keywords:** accidental falls, aged, electroencephalography, fear, gait, theta rhythm

## Abstract

**Background:**

Fear of falling is one of the most common and disabling stress responses of later life, yet how it is expressed in the brain during real walking is poorly understood. We examined whether walking under a naturalistic psychological threat, a fear‐of‐falling stressor, changes cortical oscillatory activity in older women, using mobile electroencephalography.

**Methods:**

In this within‐subject study, 30 community‐dwelling women aged 65 years or older underwent three‐dimensional gait analysis with concurrent 14‐channel electroencephalography (128 Hz) during habitual walking and after introduction of a fictional disturbing factor, a psychological, nonmotor agent that elicits fear of falling without any mechanical perturbation. Relative band power and the theta/beta ratio were compared with paired *t* tests and Cohen's dz., with sign‐flip permutation, false‐discovery‐rate correction and bootstrap confidence intervals; signal validity was confirmed by the eyes‐closed alpha increase, and head movement and gait speed served as controls.

**Results:**

Walking under threat reduced relative theta power across frontal, temporal, occipital and sensorimotor regions (*p* = 0.004–0.014; dz. around −0.5; confidence intervals excluding zero), confirmed by permutation and by seven electrodes surviving correction, while the frontal and central theta/beta ratio fell. The theta change was not accounted for by head movement, and in a smaller subgroup showed no detectable association with the change in gait speed, while gait became more cautious.

**Conclusions:**

A naturalistic fear stressor produces a measurable cortical signature during walking, positioning fear of falling as a neurobehavioural stress response with implications for the health and mobility of older women.

## 1 Introduction

Falls are among the leading causes of unintentional injury, loss of independence and mortality in older people, and fear of falling is one of their most consistent psychological determinants [1]. Recent systematic reviews estimate that about half of community‐dwelling older adults live with some degree of fear of falling, a proportion that rises among those who have fallen and in developing countries [2]. This fear is not a mere reflection of past falls. It acts as a risk factor in its own right, triggering activity restriction, physical deconditioning, social isolation and reduced quality of life, fuelling a cycle that increases the probability of further falls [3, 4].

At the motor level, fear of falling has a characteristic gait expression. Fearful older women reduce speed, shorten the step and stride and lengthen double‐support time, configuring what the literature calls cautious gait [5, 6]. Current studies confirm this profile and show that the magnitude of the changes correlates moderately to strongly with the intensity of fear, especially under more demanding balance conditions [7, 8]. This pattern is, however, ambiguous: by bringing the centre of mass closer to the base of support it protects against an immediate slip, but the loss of fluency, increased variability and greater deviation from the mature gait pattern may raise fall risk over the medium term. Although well described biomechanically, this adaptation arises from control processes that remain to be understood at the cortical level.

Human gait, once seen as a largely automatic task, increasingly recruits cortical resources whenever the environment imposes a threat to balance or additional attentional demand [9, 10]. Near‐infrared spectroscopy studies show that fear of falling is associated with greater activation and lower efficiency of the prefrontal cortex during walking [11], and recent work links balance confidence to the pattern of prefrontal activation in older adults [12, 13]. These findings support the notion that fear recruits the cortex in a specific way, but spectroscopy has limited temporal resolution and cannot capture the fast dynamics of brain rhythms.

Electroencephalography fills this gap by offering millisecond temporal resolution and, with portable systems, the feasibility of recording during movement [14, 15]. The balance and locomotion literature has identified the theta band as a central marker of postural monitoring [10]. Balance perturbations during walking and standing elicit transient increases in midfrontal theta that scale with the difficulty of the challenge [16, 17, 18, 19] and may reflect sensorimotor prediction errors [20], with distinct cortical dynamics accompanying the preparation and execution of reactive balance responses [21]. The theta/beta ratio, in turn, is an established index of attentional control and vigilance, with lower values reflecting higher alertness and greater reactivity to stress [22, 23, 24, 25, 26]. Combining these markers characterises both the magnitude and the functional direction of the cortical response.

A conceptual distinction remains underexplored. Nearly all evidence on theta and locomotion comes from real mechanical perturbations, which tend to increase midfrontal theta through prediction error [17, 18, 19, 20]. Whether a purely psychological, anticipatory threat, without any physical perturbation, produces the same response or a distinct pattern is unknown. This gap is theoretically relevant because it separates the error‐monitoring component from the anticipatory‐vigilance component of the cortical response to threat during walking.

A valid strategy to isolate this component is the fictional disturbing factor, a psychological and nonmotor agent that leads the participant to anticipate a fall risk while walking along the same track. This paradigm, validated in earlier work in three‐dimensional gait analysis, isolates the psychological component of fear from biomechanical demand and reproduces the cautious‐gait pattern [8]. Recording EEG concurrently with this paradigm characterises the cortical demand imposed by fear while keeping the nature of locomotion constant between conditions, something few studies have achieved in older women during real walking.

The aim of this study was to verify whether walking in the presence of a fear‐inducing disturbing factor used here as a naturalistic psychological stressor alters cortical oscillatory activity in older women. Rather than an exploratory approach, we adopted a directional hypothesis: that walking under threat would shift cortical activity toward a vigilance‐related pattern, characterised by reduced theta power and a lower theta/beta ratio in frontal and sensorimotor regions, in contrast to the theta increase described for mechanical perturbations.

## 2 Methods

### 2.1 Study Design and Setting

This work derives from a nonrandomised controlled trial with a within‐subject pre–post design, conducted at a university movement‐analysis laboratory. Reporting follows the applicable STROBE recommendations. The protocol complied with Resolution 466/2012 of the Brazilian National Health Council, was approved by the Research Ethics Committee of the University of Brasília—College of Ceilândia (decision number 2.109.807), and all participants gave written informed consent before any procedure. Assessments took place in a quiet, climate‐controlled room in a single session of about 2 h, with rest periods to minimise fatigue.

### 2.2 Participants

Community‐dwelling women aged 65 years or older were recruited. Inclusion criteria were female sex; age ≥ 65 years; independent walking without aids; body mass index below 30 kg/m^2^; preserved cognition, defined as a Mini‐Mental State Examination score above 24 and above 14 when adjusted for education, allowing illiterate participants; no alcohol intake in the previous 24 h and no previous contact with a gait analysis laboratory or equipment. Exclusion criteria were previous surgery of the lower limbs, pelvis or spine, diagnosis of rheumatoid arthritis, neuromuscular or neurodegenerative disease including diabetes mellitus, visual impairment and participation in other trials. Sample size was estimated a priori in G*Power 3.1.9.2, based on a one‐way analysis of variance of the overall Gait Profile Score after perturbation, indicating a need for 40 participants, with an effect size of 0.82, alpha of 0.05 and power of 0.99. Of 91 eligible women, 52 consented and 49 completed gait analysis; of these, 30 had valid EEG in both conditions and formed the analysed sample (Figure 1).

**FIGURE 1:**
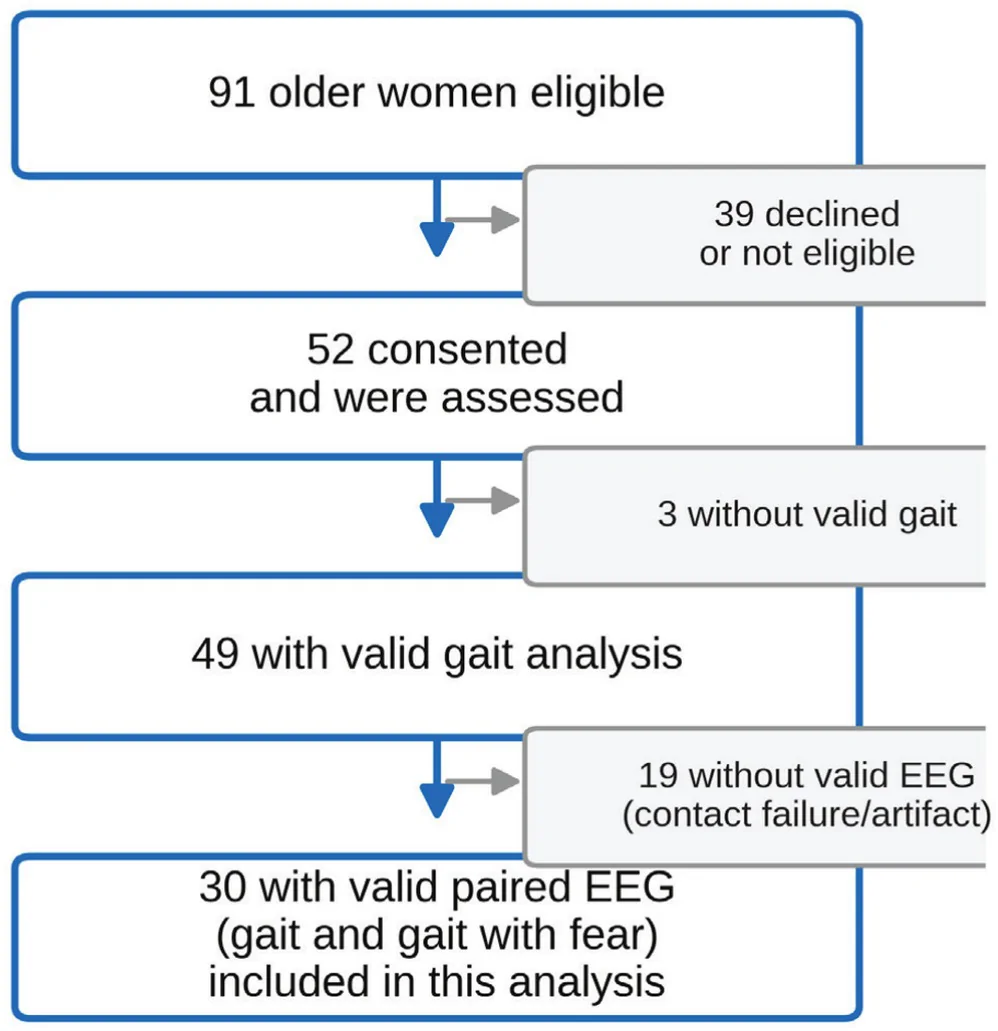
Participant flow from screening to final analysis (91 eligible; 52 consented; 49 with valid gait; 30 with valid paired EEG).

### 2.3 Clinical Assessment and Fear of Falling

Body mass and height were measured with a stadiometer scale (Filizola) and anthropometry followed the laboratory protocol, with a tape measure and calliper (Lafayette, model 01290). Cognition was screened with the Mini‐Mental State Examination [27] and physical activity with the International Physical Activity Questionnaire adapted for older adults. Two fear constructs were distinguished. Trait fear of falling, the habitual concern about falls, was quantified with the Brazilian version of the Falls Efficacy Scale International (FES‐I‐Brazil), comprising 16 items [28, 29], and served to characterise the sample, without manipulation. State fear was elicited exclusively during walking by the disturbing factor described below, which constituted the experimental manipulation.

### 2.4 Three‐Dimensional Gait Analysis and Disturbing Factor

Gait was assessed with a Vicon Nexus motion‐capture system, version 1.8.5 (Vicon Motion Systems, Oxford, UK), with eight infrared cameras and two AMTI OR6‐7 force plates, calibrated daily with a standard deviation below 0.03. Fifteen reflective markers were placed on anatomical landmarks according to the Helen Hayes protocol. Biomechanical modelling used the Conventional Gait Model, with data sampled at 120 Hz and filtered with a fourth‐order low‐pass Butterworth filter at a 10 Hz cut‐off. Each participant walked barefoot, at a self‐selected speed, over a 9 m walkway with two fixed square metal plates at the midpoint, after five familiarisation passes. Kinematic data were time‐normalised to 51 points per stride and averaged across sides. Spatiotemporal parameters and the Gait Profile Score with its nine Gait Variable Scores were computed within the Movement Analysis Profile as proposed by Baker and colleagues [30, 31], expressing in degrees the deviation from a normative database, with higher values indicating poorer quality.

Fear of falling during walking was induced by a fictional disturbing factor following the procedure validated in earlier work. After five habitual passes, participants were told that the plates fixed to the floor could vibrate strongly or deliver electrical discharges when stepped on. This is a psychological, nonmotor agent designed to create the expectation of a fall risk without any physical change to the environment or task. Only two further passes were collected after introducing the disturbing factor to avoid habituation to the fictional stimulus. The resulting conditions, habitual walking and walking with fear, preserved the same locomotor nature, differing only in the psychological component, and the disturbing factor was applied solely during walking. This procedure operationalises fear of falling as a psychogenic, affordance‐based perturbation: within a movement laboratory, gait must be challenged so as to induce the anticipation of a fall without provoking an actual fall and without devices such as partial body‐weight support, which, by conferring mechanical safety, would themselves mask the fear under study. The same perturbation profile was validated by our group in a previous three‐dimensional gait study [8]. In line with affordances theory, the disturbing factor was presented in only two passes because repeating a purely fictional threat risks extinction and new learning once the absence of any physical consequence becomes apparent; no physical stimulus was ever delivered. After the experiment, each participant was fully debriefed and informed that the vibration and electrical‐discharge warning had been fictional, and the deception entailed by the disturbing factor, together with its risk–benefit balance, was specifically reviewed and approved by the institutional Research Ethics Committee.

### 2.5 EEG Acquisition

EEG was recorded concurrently with walking using the Emotiv EPOC system (Emotiv Inc., San Francisco, USA), with 14 electrodes placed according to the international 10–20 system (AF3, F7, F3, FC5, T7, P7, O1, O2, P8, T8, FC6, F4, F8 and AF4) and two reference sensors (CMS and DRL) over parietal regions, at a 128 Hz sampling rate. The signal was acquired with TestBench software and exported in European Data Format. Standing conditions with eyes open and closed were also recorded for internal signal validation through alpha‐rhythm reactivity.

### 2.6 EEG Processing

Processing was performed in Python (pyEDFlib, NumPy and SciPy). The 14 EEG channels were filtered with a zero‐phase fourth‐order Butterworth band‐pass between 1 and 45 Hz; channels with null or saturated signal were discarded. Power spectral density was estimated by Welch's method (Hann window, 256 samples, 50% overlap). Relative band power was the fraction of total power between 1 and 30 Hz in delta (1–4 Hz), theta (4–8 Hz), alpha (8–13 Hz) and beta (13–30 Hz), an option that reduces the influence of global amplitude differences between conditions. Values were averaged per participant across valid epochs of each condition and grouped into frontal, temporal, parietal, occipital and sensorimotor regions, with the theta/beta ratio computed per region. Head movement, used in artefact‐control analyses, was quantified from the variability of the device gyroscope channels in each epoch.

### 2.7 Statistical Analysis

Habitual and fear conditions were compared by paired *t* test with Cohen's dz., whose 95% confidence intervals were obtained by bootstrap with 5000 resamples. A sign‐flip permutation test with 10 000 permutations confirmed the main effect without distributional assumptions. Comparisons were corrected for multiple comparisons by the Benjamini‐Hochberg procedure in two complementary families: across the 14 electrodes within theta, to define topography, and across the 20 region‐by‐band combinations, to verify spectral specificity. To examine brain‐gait coupling and rule out a speed effect, the between‐condition theta change was correlated, per participant, with the change in gait speed, double support and Gait Profile Score in the subgroup with paired EEG and gait, using Spearman correlation. As a further artefact‐control step, a sensitivity analysis applied independent component analysis (FastICA) to the 14 channels of each recording and removed components whose time course correlated with the gyroscope signal (absolute r above 0.40), after which the theta contrast was repeated on the reconstructed signal. Signal validity was confirmed by paired comparison of alpha power between eyes open and closed. Significance was set at 0.05; analyses used SciPy, statsmodels and scikit‐learn. Because myogenic activity is broadband and predominates above 20–30 Hz [32, 33], muscle contamination was further limited by the Common‐Mode‐Sense and Driven‐Right‐Leg reference of the headset, which cancels common‐mode interference and shared scalp noise [34], by the band‐pass filtering and independent component analysis described above, following practice established for mobile and consumer‐grade recordings [35, 36], and by confining interpretation to theta; activity in beta, the highest band analysed and the closest to the myogenic range, was examined as a control.

## 3 Results

### 3.1 Signal Validation

In standing recordings, relative alpha power was higher with eyes closed than open in all tested regions, most markedly occipital (difference 0.053; dz. 0.64; *p* = 0.007), followed by frontal (dz 0.50; *p* = 0.028) and parietal (dz 0.45; *p* = 0.047) (Figure 2). This reproduces the classical occipital alpha increase on eye closure and confirms that the system captured physiological cortical activity.

**FIGURE 2:**
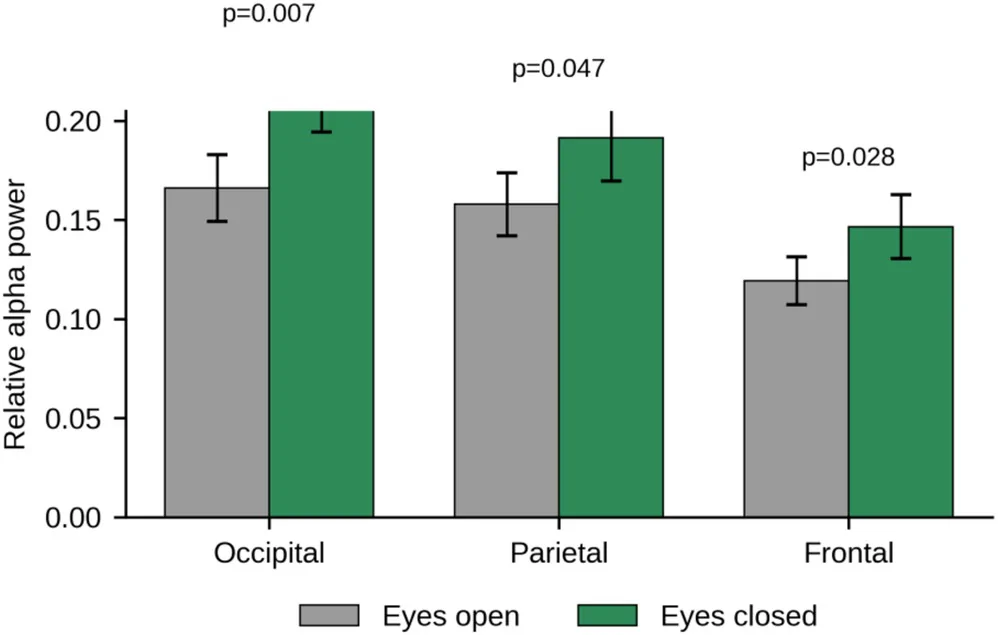
Relative alpha power in standing with eyes open versus closed (*n* = 22). The alpha increase with eyes closed confirms EEG signal validity. Bars show mean and standard error.

### 3.2 Biomechanical Response to the Disturbing Factor

Before examining the cortical response, the behavioural effect of the disturbing factor was characterised in the cohort (*n* = 49). Exposure produced a clear cautious‐gait signature, with reduced speed, step and stride length and cadence, and increased stride time, double support and stance phase (Table 1). Effects were moderate to large, led by walking speed (dz = −0.95) and stride length (dz = −0.90), whereas single support was unchanged, indicating a specific conservative strategy bringing the centre of mass closer to the base of support (Figure 3).

**TABLE 1:** Spatiotemporal gait parameters without and with the disturbing factor (*n* = 49).

Parameter	Gait	With fear	dz	*p*
Walking speed (m/s)	0.98 ± 0.18	0.85 ± 0.23	−0.95	< 0.001
Stride length (m)	1.06 ± 0.15	0.95 ± 0.20	−0.90	< 0.001
Cadence (steps/min)	109.8 ± 9.9	106.1 ± 12.4	−0.40	0.008
Stride time (s)	1.11 ± 0.11	1.16 ± 0.16	0.41	0.006
Double support (s)	0.24 ± 0.07	0.31 ± 0.14	0.56	< 0.001
Single support (s)	0.44 ± 0.03	0.44 ± 0.07	−0.01	0.949
Stance phase (% cycle)	61.7 ± 2.6	63.3 ± 3.6	0.54	< 0.001

*Note:* Values are mean ± standard deviation. Gait, habitual walking; With fear, walking in the presence of the fictional disturbing factor. Conditions were compared with paired *t* tests; dz. is Cohen's standardised effect size for paired samples, with negative values indicating a reduction under fear. *p* is two‐tailed and unadjusted, with significance set at *p* < 0.05. Values as mean ± standard deviation; Cohen's dz and paired *t* test p.

Abbreviation: SD, standard deviation.

**FIGURE 3:**
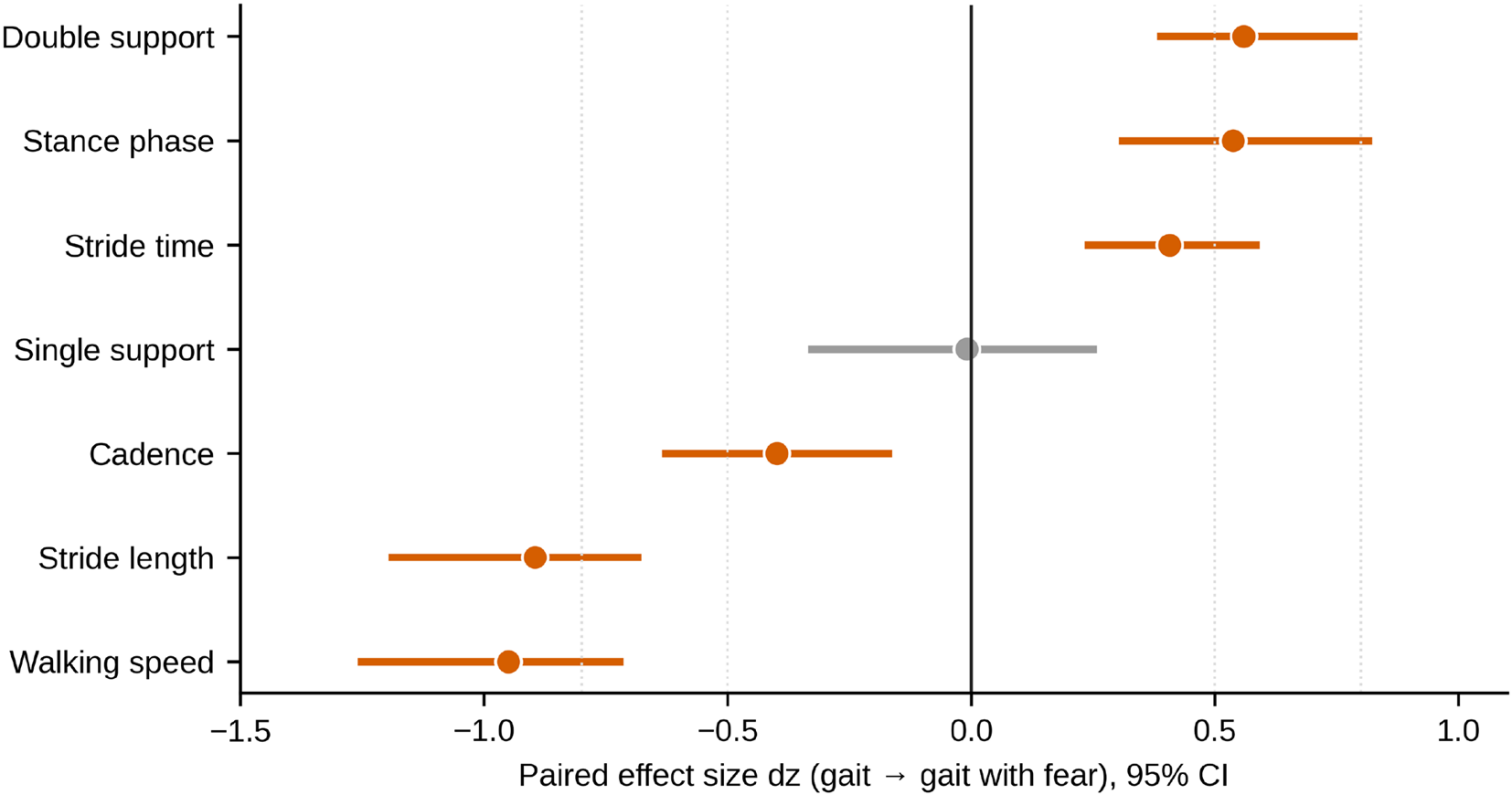
Paired effect size (dz, with bootstrap 95% CI) of spatiotemporal variables, from gait to gait with fear (*n* = 49). Orange points indicate significant effects. Walking speed and stride length show the largest reductions, while single support is unchanged.

The overall Gait Profile Score worsened consistently under the disturbing factor (pooled increase from about 8.9–9.9 degrees), with the largest Gait Variable Score deteriorations at knee flexion/extension and ankle dorsi/plantarflexion, plus hip ab/adduction.

### 3.3 Theta Reduction When Walking With Fear

Walking with the disturbing factor was associated with a broad reduction in relative theta power, significant in the frontal (dz = −0.49; *p* = 0.012), temporal (dz = −0.56; *p* = 0.004), sensorimotor (dz = −0.51; *p* = 0.009) and occipital (dz = −0.47; *p* = 0.014) regions, with a nonsignificant trend parietally (*p* = 0.127) (Figure 4, Table 2). The effect was robust to distribution‐free approaches: a sign‐flip permutation test confirmed the frontal reduction (*p* = 0.006), and the effect‐size confidence intervals excluded zero for both the global mean (dz = −0.51; 0.20–0.87) and the frontal region (dz = −0.49; 0.19–0.88).

**FIGURE 4:**
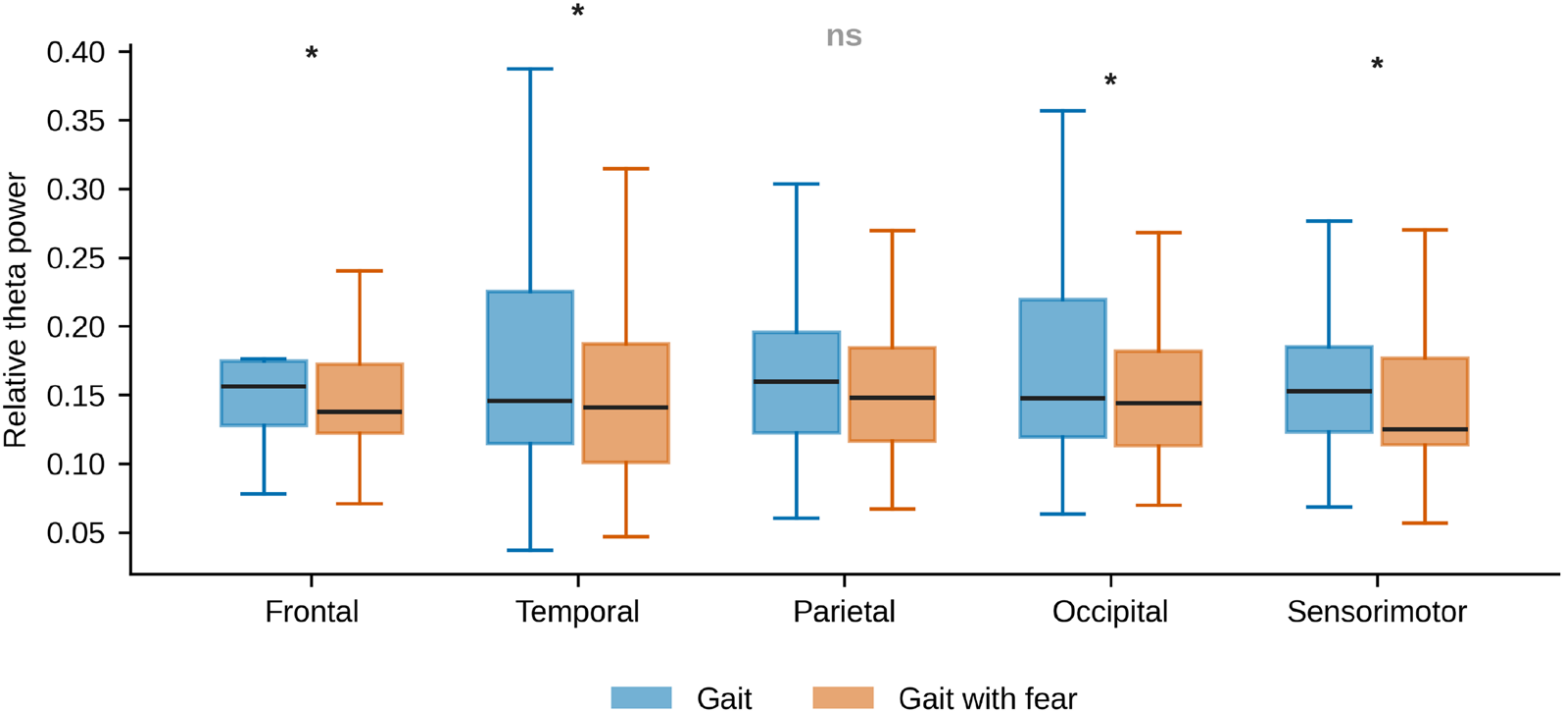
Relative theta power during gait and gait with fear, by region (*n* = 30). Boxes show median and quartiles; whiskers show dispersion. The asterisk indicates a significant paired difference. Full regional values and effect sizes are in Table 2.

**TABLE 2:** Relative theta power during gait and gait with fear, by region (*n* = 30).

Region	Gait	With fear	Difference [95% CI]	dz [95% CI]	*p*	*q*
Frontal	0.173 ± 0.069	0.158 ± 0.064	−0.015 [−0.026; −0.005]	−0.49 [−0.89; −0.18]	0.012	0.018
Temporal	0.172 ± 0.087	0.156 ± 0.080	−0.016 [−0.026; −0.006]	−0.56 [−0.96; −0.23]	0.004	0.018
Sensorimotor	0.167 ± 0.066	0.150 ± 0.064	−0.017 [−0.030; −0.006]	−0.51 [−0.88; −0.21]	0.009	0.018
Occipital	0.175 ± 0.077	0.159 ± 0.065	−0.015 [−0.026; −0.004]	−0.47 [−0.94; −0.13]	0.014	0.018
Parietal	0.174 ± 0.075	0.164 ± 0.073	−0.010 [−0.022; 0.002]	−0.29 [−0.70; 0.06]	0.127	0.127

*Note:* Gait and With fear values are mean ± standard deviation of relative theta power (theta‐band power as a proportion of total 4–45 Hz power). Difference is the within‐subject change (With fear minus Gait) and dz. is Cohen's effect size for paired samples, each shown with a bootstrap 95% confidence interval. Conditions were compared with paired *t* tests; *p* is two‐tailed and *q* is the Benjamini and Hochberg false‐discovery‐rate‐adjusted value across the five regions, with significance set at *q* < 0.05. Mean ± SD; difference and dz with bootstrap 95% CI; raw *p* and FDR‐corrected *q* (five‐region family).

Abbreviations: CI, confidence interval; FDR, false discovery rate; SD, standard deviation.

Electrode‐wise, seven channels remained significant after FDR within theta, configuring a temporo‐fronto‐occipital distribution: T8 (dz = −0.61), FC5 (dz = −0.56), F8 (dz = −0.56), O2 (dz = −0.53), T7 (dz = −0.46) and the prefrontal AF3 and AF4 (dz = −0.45), all with corrected *p* ≤ 0.042 (Figure 5). Across the broader 20‐test family the four regional theta reductions remained the largest effects, while no other band approached significance, indicating that the effect is spectrally specific to theta and spatially distributed.

**FIGURE 5:**
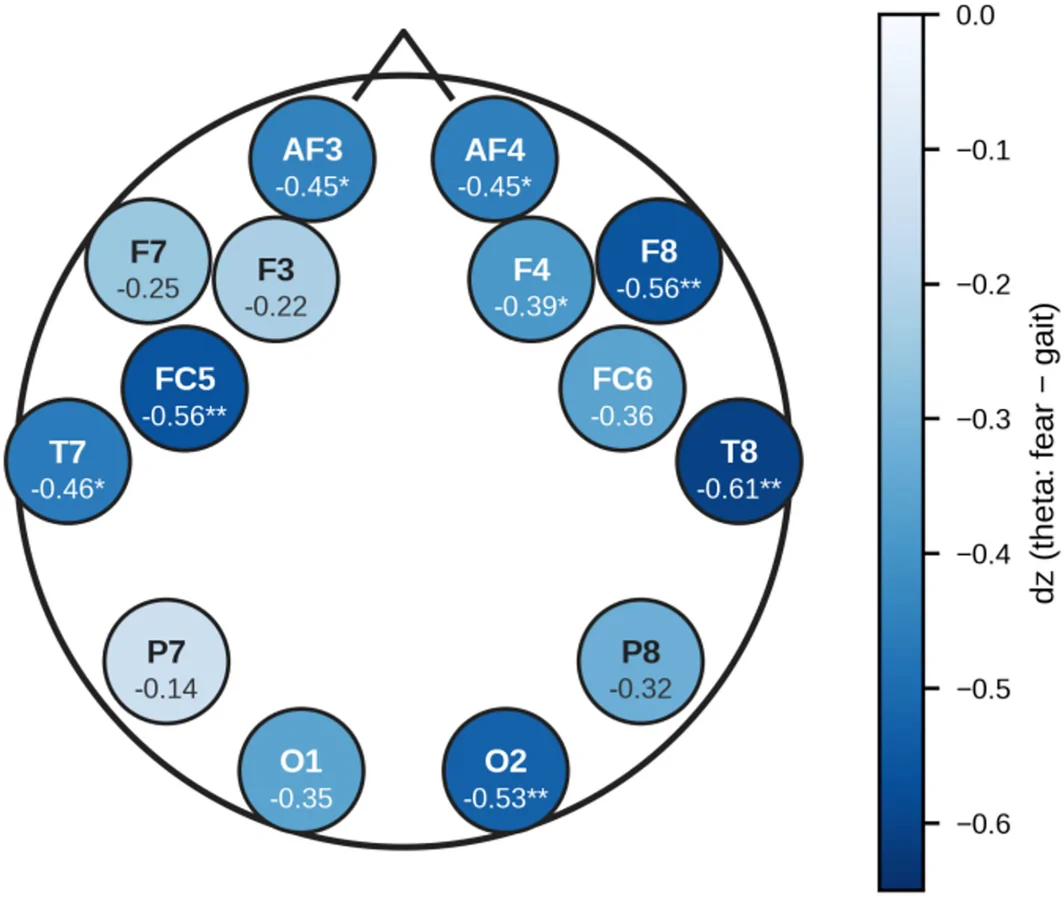
Topographic distribution of the effect of fear on theta power (effect size dz). Darker shades indicate larger reductions. *p < 0.05; **p < 0.01 (paired, *n* = 30).

### 3.4 Theta/Beta Ratio

The theta/beta ratio decreased with fear in all regions, reaching significance frontally, temporally and in the sensorimotor region, with a trend parietally and occipitally (Table 3). This decrease, driven by lower theta with a slight beta increase, independently supports a shift toward a vigilance state.

**TABLE 3:** Theta/beta ratio during gait and gait with fear, by region (*n* = 30).

Region	Gait	With fear	dz	*p*
Frontal	1.11	0.93	−0.39	0.040
Temporal	1.09	0.85	−0.41	0.031
Sensorimotor	1.03	0.80	−0.42	0.028
Parietal	1.03	0.86	−0.37	0.053
Occipital	1.05	0.85	−0.32	0.092

*Note:* Values are the mean theta/beta ratio (relative theta power divided by relative beta power) in each condition (*n* = 30); a lower ratio reflects a relatively smaller theta contribution and is commonly read as a marker of greater attentional or cognitive‐control engagement. Conditions were compared with paired *t* tests; dz. is Cohen's standardised effect size for paired samples, with negative values indicating a decrease under fear. *p* is two‐tailed; per‐region values are unadjusted and parallel the false‐discovery‐rate analysis of relative theta power in Table 2. Significance was set at *p* < 0.05.

### 3.5 Movement‐Artefact Control

As recording occurred during walking, it was necessary to rule out that the theta reduction merely reflected greater movement under fear. Head movement was indeed larger with fear, yet theta change did not follow the artefact in the expected direction. The correlation between theta change and movement change was positive (ρ = 0.40; *p* = 0.029), meaning that participants who moved more showed smaller theta reductions, the opposite of an artefact. Decisively, when theta change was regressed on movement change, the intercept (theta change at zero movement change) remained negative and significant (*p* = 0.007), while the slope was not significant (Figure 6). The theta reduction is therefore not explained by movement, and the slight beta increase reinforces this, since movement artefact tends to inflate low rather than high frequencies. The finding also held in a sensitivity analysis in which independent components correlated with the gyroscope were removed before recomputing the spectra: frontal theta still decreased with fear (0.186–0.154; dz. = −0.41; *p* = 0.031).

**FIGURE 6:**
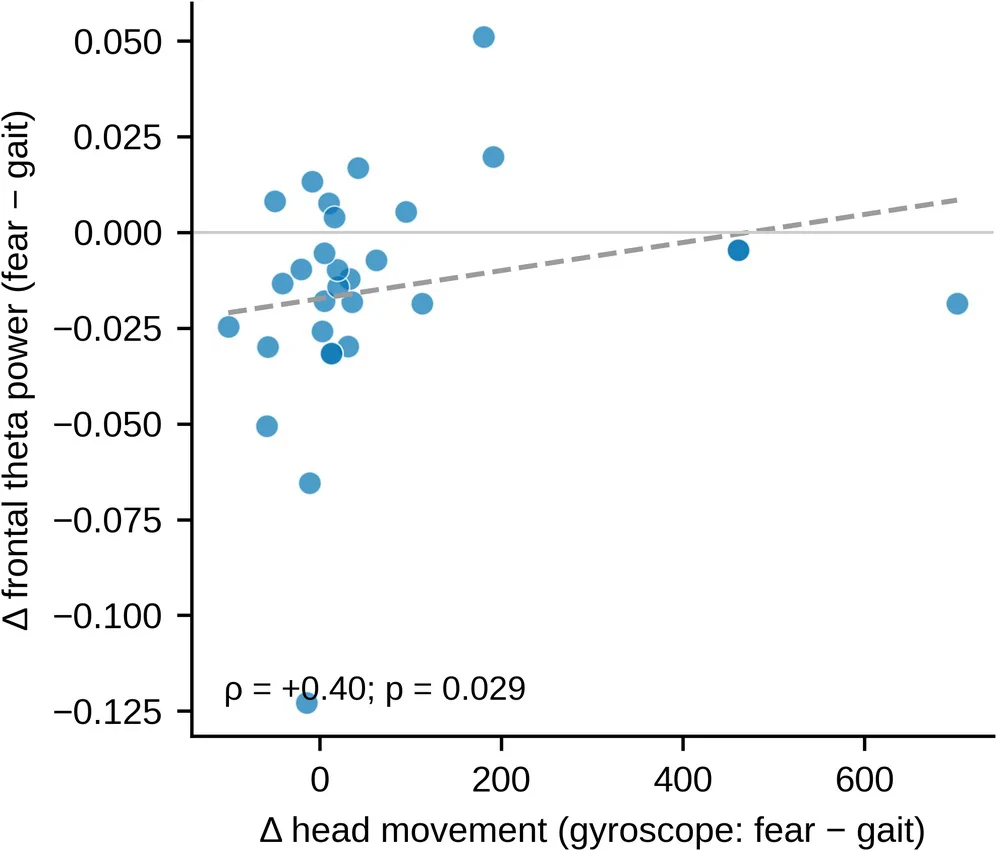
Change in frontal theta power versus change in head movement between conditions (fear minus gait; *n* = 30). The absence of a negative relationship argues against the theta reduction stemming from movement artefact.

### 3.6 Brain‐Gait Relationship

In the subgroup with paired EEG and three‐dimensional gait in the same session (*n* = 20), the magnitude of theta reduction was tested against the magnitude of biomechanical change. Theta change showed no detectable correlation with the change in gait speed (ρ = −0.03; *p* = 0.90; Figure 7); given the small subgroup, this is best read as a failure to detect an association rather than as evidence that cortical suppression is independent of walking speed. Correlations with double‐support and Gait Profile Score change were also nonsignificant (ρ ≈0.21; *p* > 0.37). Thus, although the cortical and biomechanical responses co‐occur under the same threat, we did not detect linear coupling between their magnitudes at this sample size; this result is inconclusive and does not establish that the two components are independent.

**FIGURE 7:**
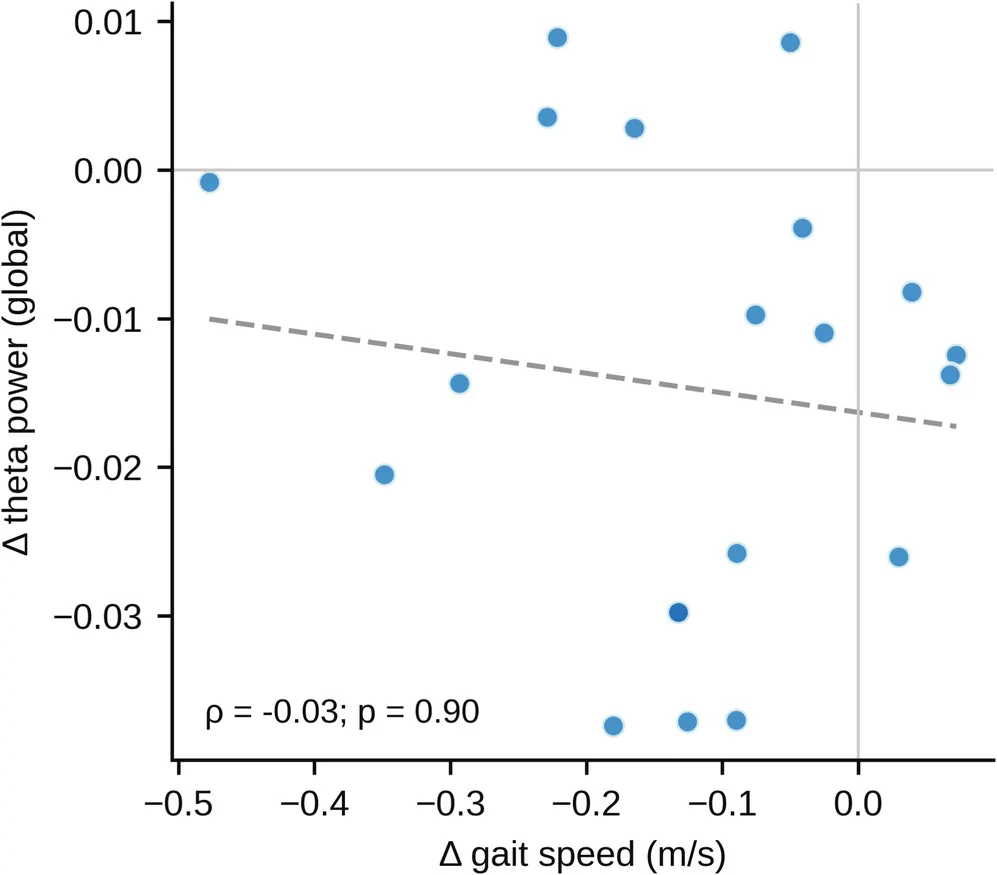
Change in global theta power versus change in gait speed between conditions (fear minus gait; *n* = 20). No association was detected between the two changes; given the small subgroup, this does not by itself establish that the theta reduction is independent of the speed reduction.

### 3.7 Trait Fear

In an exploratory analysis, the magnitude of the cortical response was tested against trait fear of falling (FES‐I). In the subset with paired EEG and an available score (*n* = 17), the correlation was not significant (ρ = 0.22; *p* = 0.40). This should be interpreted cautiously, as the small number strongly limits power and does not allow concluding the absence of a relationship.

## 4 Discussion

The main finding is that an acute fear‐of‐falling context during real walking suppresses cortical theta power and reduces the theta/beta ratio in older women, despite the absence of any mechanical perturbation. The effect was spatially broad, statistically robust and independent of movement, and validation by the occipital alpha increase on eye closure supports the recording, a particularly important safeguard for portable EEG during locomotion.

The direction of the effect is the most original aspect and warrants careful interpretation. The mechanical‐perturbation literature links midfrontal theta to postural monitoring and sensorimotor prediction error, with transient theta increases proportional to challenge intensity [17, 18, 19, 20]. Our finding goes the opposite way, and this apparent contradiction is precisely what distinguishes the paradigm. When the threat is mechanical and real, the cortex must process the error between predicted and executed movement, raising theta. When the threat is psychological and anticipatory, with no physical perturbation, there is no sensorimotor error to correct; instead, the system appears to leave an internal‐monitoring mode and enter a continuous sensory‐vigilance mode, in which relative theta falls and beta tends to rise. This distinction between prediction error, in physical perturbation, and anticipatory vigilance, in psychological threat, is consistent with the lower theta/beta ratio, an established marker in which lower values indicate greater attentional reactivity to stress [22, 23, 24], and dialogues with spectroscopy studies describing lower prefrontal efficiency under fear of falling during walking [11]. The distributed nature of the effect is compatible with a global state shift rather than a single error‐processing focus.

The interpretation gains strength when read alongside the biomechanical response in the same paradigm. The disturbing factor produced a typical cautious gait, with reduced speed, step and stride and increased double support, and raised the Gait Profile Score, indicating objective worsening of movement quality [8, 30]. The largest Gait Variable Score deteriorations occurred at the knee and ankle, distal joints responsible for propulsion and fine support control, precisely those most demanding of predictive control during locomotion. The co‐occurrence, under the same threat, of a cortical hypervigilance signature and a slower, shorter, more deviant gait is consistent with cortical reorganisation and a conservative motor strategy being coupled manifestations of one defensive response. These findings are consistent with a shift toward a vigilance‐dominant locomotor state, in which the protective gait, while reducing the immediate slip risk, increases variability and deviation from the mature pattern and may, over the medium term, raise fall risk. We summarise this chain in a neurobehavioural model (Figure 8); being interpretive, its arrows are hypotheses to be tested, not established causal links.

**FIGURE 8:**
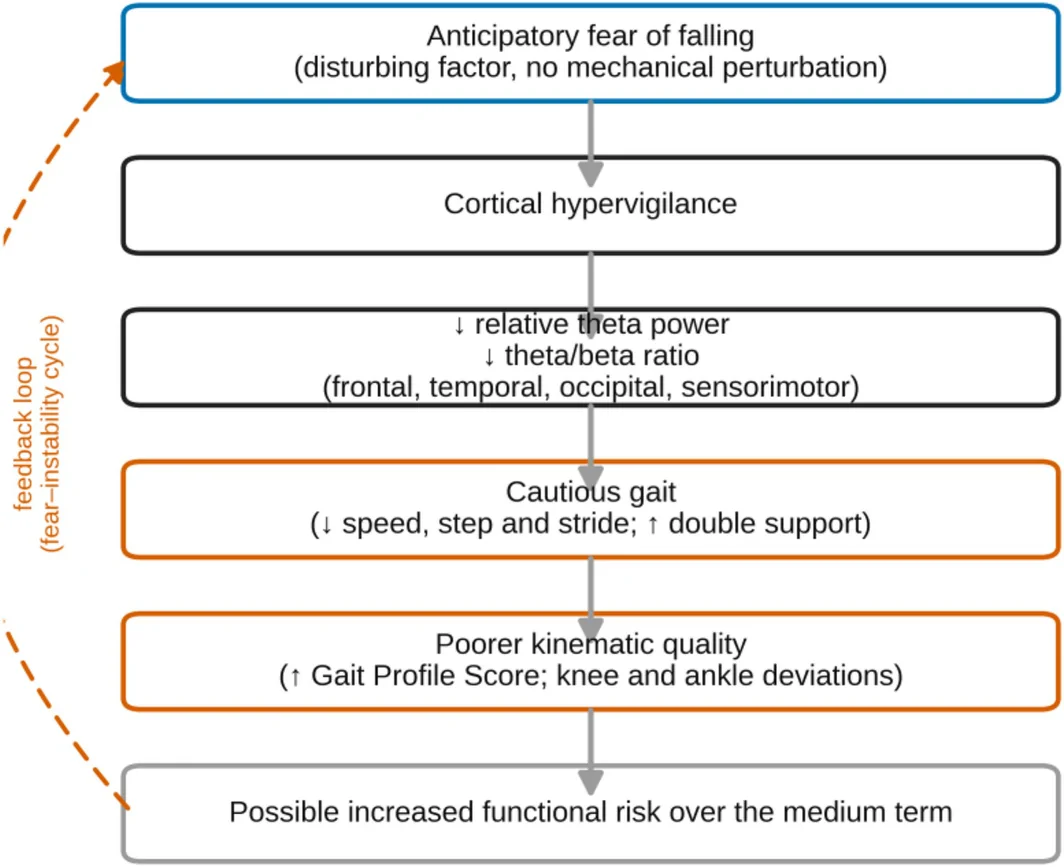
Proposed neurobehavioural model for the response to fall threat during walking. Arrows represent hypothetical relationships, to be tested in longitudinal and intervention studies.

Confidence in these findings rests on safeguards uncommon in mobile EEG studies. The main effect was confirmed by permutation and by effect‐size confidence intervals excluding zero; spectral specificity was shown by FDR correction across two comparison families, with the effect restricted to theta; the topography survived per‐electrode FDR within theta and the main threat to this type of recording, movement artefact, was addressed directly, as theta reduction persisted when movement change was controlled and the movement‐theta relationship ran opposite to that expected of an artefact. In a smaller subgroup, theta change was not significantly associated with the change in gait speed; because this analysis was underpowered, it indicates a failure to detect coupling rather than a demonstration that cortical suppression is independent of walking speed. Together, these elements are consistent with a genuine physiological effect rather than an epiphenomenon of recording during gait, although they do not exclude every nonneural contribution.

Clinically, the finding connects the behavioural and neural planes and opens concrete possibilities. The cortical state could serve as an objective, sensitive outcome in rehabilitation programmes targeting fear of falling, complementing self‐report and gait parameters; as a marker of response to interventions; and as a means to identify older women with an exaggerated defensive cortical response, potentially more vulnerable. The feasibility of recording with a low‐cost portable system during real walking with a standardised fear stimulus [15, 37] favours integration with emerging approaches such as threat‐controlled gait training, virtual reality, dual‐task paradigms, biofeedback and neuromodulation, in which theta suppression could act as a target or efficacy indicator.

These results also help frame fear of falling as a neurobehavioural, and not merely psychological, phenomenon. A measurable cortical response during threatened locomotion supports the view that fear actively modulates cortical motor control, with implications for understanding the mechanisms linking fear to increased fall risk.

### 4.1 Limitations

Several limitations should be weighed. The cross‐sectional design precludes causal inference. The 14‐electrode system has limited spatial coverage and no source localisation, so the topographic reading should be interpreted at the sensor level. The relationship between cortical response magnitude and trait fear was only exploratory and clearly underpowered (*n* = 17), and the brain‐gait analysis was possible in only 20 women; the nonsignificant correlations in these subgroups represent a failure to detect associations rather than evidence that the corresponding relationships are absent, and confirmation in larger samples remains necessary. Gait speed decreased under fear and could modulate cortical activity; although theta change showed no detectable association with speed change, was not explained by head movement, survived a gyroscope‐guided independent component analysis and was confined to theta, we cannot fully exclude a contribution of slower walking, and larger studies should include speed as a covariate in mixed models. The overall sample, adequate for the medium effects and paired design, nonetheless limits subgroup analyses and the detection of small effects.

Some further considerations qualify the interpretation. First, the two conditions were recorded in a fixed order, habitual walking before walking with fear, and no immediate subjective rating of fear or anxiety, for example a single‐item visual‐analogue scale, was obtained as a manipulation check; the fear state was inferred from the pre to postperturbation change in gait, following the affordance‐based paradigm validated previously [8]. Counterbalancing and repeated fear exposures were deliberately avoided, because the disturbing factor is a purely psychogenic, nonphysical threat that would be extinguished by repetition; we nonetheless acknowledge that the fixed order leaves open some influence of time‐on‐task or habituation, and that a brief postcondition fear rating would strengthen future studies. Second, the primary outcome was relative band power, chosen deliberately to control for global amplitude and for differences between individuals and electrodes; because relative power is compositional, a fall in relative theta is necessarily accompanied by changes in other bands, yet the reduction was spectrally specific, surviving false‐discovery‐rate correction within theta and across the 20 region‐by‐band comparisons, while beta showed only a small, nonsignificant change, so the pattern is not a simple redistribution driven by a single band, and confirmation in absolute units, that is absolute theta power together with the total 1–30 Hz power per condition, is a natural next step rather than a threat to the present result. Third, the fear condition comprised two passes against five in the habitual condition; each acquisition yields six analysis windows, so about 12 windows contributed to the fear condition and about 30 to the habitual condition per participant, an asymmetry inherent to the paradigm, since repeating the fictional threat would induce learning; 12 windows remain adequate for a stable Welch estimate, and these counts are now reported. Fourth, scalp electroencephalography during walking is vulnerable to myogenic activity of the neck and jaw, which is broadband and predominates above 20–30 Hz [32, 33]; this was mitigated by the Common‐Mode‐Sense and Driven‐Right‐Leg reference of the device [34], by band‐pass filtering and independent component analysis following practice established for mobile and consumer‐grade EEG [35, 36], and by confining interpretation to theta, below the range in which muscle activity dominates; consistent with this, no band above theta increased significantly, the opposite of the broadband elevation expected from muscle contamination. Finally, participants were cognitively intact women with a body mass index below 30 kg/m^2^, without diabetes, neuromuscular or neurodegenerative disease and without prior laboratory fall testing; this narrow profile was a deliberate choice to isolate the psychogenic component of fear of falling from physical and cognitive confounders, but it limits generalisability to the more comorbid or cognitively impaired individuals often seen in clinical practice, and fear of falling is not tied to comorbidity alone but also to ageing itself and to gerontological and public‐health framing.

## 5 Conclusion

Walking in the presence of a fear‐inducing disturbing factor reduces relative cortical theta power and the theta/beta ratio in older women, a pattern consistent with heightened vigilance during threatened locomotion and not accounted for by head movement; a smaller subgroup analysis did not detect an association with gait speed, although it was underpowered to exclude one. Mobile EEG proved valid and sensitive to capture this response, supporting its use to investigate and monitor the cortical demand associated with fear of falling, with potential application in fall‐prevention programmes.

## Funding

The authors have nothing to report.

## Ethics Statement

The study was approved by the institutional Research Ethics Committee (approval number 2.109.807) and was conducted in accordance with the Declaration of Helsinki and Resolution 466/2012 of the Brazilian National Health Council. The deception entailed by the disturbing factor and its risk–benefit balance were specifically evaluated and approved, and all participants were debriefed after the experiment.

## Consent

All participants provided written informed consent before any procedure.

## Conflicts of Interest

The authors declare no conflicts of interest.

## Data Availability

The data that support the findings of this study are available on request from the corresponding author. The data are not publicly available due to privacy or ethical restrictions.

## References

[1] World Health Organization , Step Safely: Strategies for Preventing and Managing Falls Across the Life‐Course (WHO, 2021).

[2] N. Salari , N. Darvishi , M. Ahmadipanah , et al., “The Global Prevalence of and Risk Factors for Fear of Falling Among Older Adults: A Systematic Review and Meta‐Analysis,” BMC Geriatrics 24 (2024): 325. 10.1186/s12877-024-04882-w PMC10998426 38580924

[3] C. Whitmore , S. Neil‐Sztramko , S. Grenier , et al., “Factors Associated With Anxiety and Fear of Falling in Older Adults: A Rapid Systematic Review of Reviews,” PLoS One 19, no. 12 (2024): e0315185. 10.1371/journal.pone.0315185 PMC11654959 39693333

[4] D. Schoene , C. Heller , Y. N. Aung , C. C. Sieber , W. Kemmler , and E. Freiberger , “A Systematic Review on the Influence of Fear of Falling on Quality of Life in Older People: Is There a Role for Falls?,” Clinical Interventions in Aging 14 (2019): 701–719. 10.2147/CIA.S197857 PMC6514257 31190764

[5] A. Brachman , V. Hadyk , A. Kamieniarz‐Olczak , and A. Nawrat‐Szołtysik , “Examining the Role of Fear of Falling on Gait Parameters and Short‐Term Gait Adaptation in Older Adults,” Journal of Clinical Medicine 14 (2025): 8311, 10.3390/jcm14238311. PMC12693663 41375614

[6] I. Teles , J. Moreira , and A. S. P. Sousa . “Association Between the History of Fall and the Fear of Falling on Stair‐Descent and Gait‐Transition Spatiotemporal Parameters and Lower‐Limb Kinematics in Older Adults,” Applied Sciences 15, no. 12 (2025): 6689, 10.3390/app15126689.

[7] A. Suri , Z. L. Hubbard , J. VanSwearingen , et al., “Fear of Falling in Community‐Dwelling Older Adults: What Their Gait Acceleration Pattern Reveals,” Computer Methods and Programs in Biomedicine 244 (2024): 108001, 10.1016/j.cmpb.2023.108001. PMC11411449 38199138

[8] G. A. S. Bueno , F. M. Gervásio , D. M. Ribeiro , A. C. Martins , T. V. Lemos , and R. L. de Menezes , “Fear of Falling Contributing to Cautious Gait Pattern in Women Exposed to a Fictional Disturbing Factor: A Non‐Randomized Clinical Trial,” Frontiers in Neurology 10 (2019): 283. 10.3389/fneur.2019.00283 PMC6445048 30972013

[9] D. J. Clark , “Automaticity of Walking: Functional Significance, Mechanisms, Measurement and Rehabilitation Strategies,” Frontiers in Human Neuroscience 9 (2015): 246. 10.3389/fnhum.2015.00246 PMC4419715 25999838

[10] S. Stuart , R. Vitorio , R. Morris , D. N. Martini , P. C. Fino , and M. Mancini , “Cortical Activity During Walking and Balance Tasks in Older Adults and in People With Parkinson's Disease: A Structured Review,” Maturitas 113 (2018): 53–72. 10.1016/j.maturitas.2018.04.011 PMC6448561 29903649

[11] R. Holtzer , R. Kraut , M. Izzetoglu , and S. Ye , “The Effect of Fear of Falling on Prefrontal Cortex Activation and Efficiency During Walking in Older Adults,” GeroScience 41, no. 1 (2019): 89–100. 10.1007/s11357-019-00056-4 PMC6423209 30737727

[12] J. Y. Min , B. Y. Choi , S. W. Ryoo , et al., “Greater Prefrontal Cortical Activation Is Associated With Higher Balance Confidence in Older Adults,” GeroScience 48 (2025): 3137–3150, 10.1007/s11357-025-02003-y. PMC12972348 41219663

[13] T. Hadjistavropoulos , K. Delbaere , and T. D. Fitzgerald , “Reconceptualizing the Role of Fear of Falling and Balance Confidence in Fall Risk,” Journal of Aging and Health 23 (2011): 3–23. 10.1177/0898264310378039 20852012

[14] J. T. Gwin , K. Gramann , S. Makeig , and D. P. Ferris , “Electrocortical Activity Is Coupled to Gait Cycle Phase During Treadmill Walking,” NeuroImage 54, no. 2 (2011): 1289–1296. 10.1016/j.neuroimage.2010.08.066 20832484

[15] A. Lau‐Zhu , M. P. H. Lau , and G. McLoughlin , “Mobile EEG in Research on Neurodevelopmental Disorders: Opportunities and Challenges,” Developmental Cognitive Neuroscience 36 (2019): 100635. 10.1016/j.dcn.2019.100635 PMC6534774 30877927

[16] A. M. Payne and L. H. Ting , “Worse Balance Is Associated With Larger Perturbation‐Evoked Cortical Responses in Healthy Young Adults,” Gait & Posture 80 (2020): 324–330. 10.1016/j.gaitpost.2020.06.018 PMC7436194 32593102

[17] S. M. Peterson and D. P. Ferris , “Differentiation in Theta and Beta Electrocortical Activity Between Visual and Physical Perturbations to Walking and Standing Balance,” eNeuro 5, no. 4 (2018): 1–20. 10.1523/ENEURO.0207-18.2018 PMC6088363 30105299

[18] M. Stokkermans , T. Solis‐Escalante , M. X. Cohen , and V. Weerdesteyn , “Midfrontal Theta Dynamics Index the Monitoring of Postural Stability,” Cerebral Cortex 33, no. 7 (2023): 3454–3466, 10.1093/cercor/bhac283. PMC10068289 36066445

[19] A. R. Sipp , J. T. Gwin , S. Makeig , and D. P. Ferris , “Loss of Balance During Balance‐Beam Walking Elicits a Multifocal Theta‐Band Electrocortical Response,” Journal of Neurophysiology 110 (2013): 2050–2060. 10.1152/jn.00744.2012 PMC3841925 23926037

[20] N. A. Jacobsen and D. P. Ferris , “Electrocortical Theta Activity May Reflect Sensory Prediction Errors During Adaptation to a Gradual Gait Perturbation,” PeerJ 12 (2024): e17451, 10.7717/peerj.17451. PMC11162180 38854799

[21] T. Solis‐Escalante , J. van der Cruijsen , D. de Kam , J. van Kordelaar , V. Weerdesteyn , and A. C. Schouten , “Cortical Dynamics During Preparation and Execution of Reactive Balance Responses With Distinct Postural Demands,” NeuroImage 188 (2019): 557–571. 10.1016/j.neuroimage.2018.12.045 30590120

[22] J. F. Cavanagh and M. J. Frank , “Frontal Theta as a Mechanism for Cognitive Control,” Trends in Cognitive Sciences 18 (2014): 414–421. 10.1016/j.tics.2014.04.012 PMC4112145 24835663

[23] P. Putman , B. Verkuil , E. Arias‐Garcia , I. Pantazi , and C. van Schie , “EEG Theta/Beta Ratio as a Potential Biomarker for Attentional Control and Resilience Against the Deleterious Effects of Stress on Attention,” Cognitive, Affective, & Behavioral Neuroscience 14 (2014): 782–791. 10.3758/s13415-013-0238-7 24379166

[24] A. Angelidis , W. van der Does , L. Schakel , and P. Putman , “Frontal EEG Theta/Beta Ratio as an Electrophysiological Marker for Attentional Control and Its Relationship With Anxiety,” Biological Psychology 121 (2016): 49–52. 10.1016/j.biopsycho.2016.09.008 27697551

[25] A. Angelidis , M. Hagenaars , D. van Son , W. van der Does , and P. Putman , “Do Not Look Away! Spontaneous Frontal EEG Theta/Beta Ratio as a Marker for Cognitive Control Over Attention to Mild and High Threat,” Biological Psychology 135 (2018): 8–17. 10.1016/j.biopsycho.2018.03.002 29518523

[26] T. S. Wang , S. S. Wang , C. L. Wang , and S. B. Wong , “Theta/Beta Ratio in EEG Correlated With Attentional Capacity Assessed by the Conners Continuous Performance Test in Children With ADHD,” Frontiers in Psychiatry 14 (2023): 1305397. 10.3389/fpsyt.2023.1305397 PMC10834743 38312917

[27] M. F. Folstein , S. E. Folstein , and P. R. McHugh , “Mini‐Mental State: A Practical Method for Grading the Cognitive State of Patients for the Clinician,” Journal of Psychiatric Research 12 (1975): 189–198. 10.1016/0022-3956(75)90026-6 1202204

[28] L. Yardley , N. Beyer , K. Hauer , G. Kempen , C. Piot‐Ziegler , and C. Todd , “Development and Initial Validation of the Falls Efficacy Scale‐International (FES‐I),” Age and Ageing 34 (2005): 614–619. 10.1093/ageing/afi196 16267188

[29] F. F. O. Camargos , R. C. Dias , J. M. D. Dias , and M. T. F. Freire , “Cross‐Cultural Adaptation and Psychometric Properties of the FES‐I‐Brazil,” Revista Brasileira de Fisioterapia 14 (2010): 237–243. 20730369

[30] R. Baker , J. L. McGinley , M. H. Schwartz , et al., “The Gait Profile Score and Movement Analysis Profile,” Gait & Posture 30 (2009): 265–269. 10.1016/j.gaitpost.2009.05.020 19632117

[31] M. H. Schwartz and A. Rozumalski , “The Gait Deviation Index: A New Comprehensive Index of Gait Pathology,” Gait & Posture 28 (2008): 351–357. 10.1016/j.gaitpost.2008.05.001 18565753

[32] I. I. Goncharova , D. J. McFarland , T. M. Vaughan , and J. R. Wolpaw , “EMG Contamination of EEG: Spectral and Topographical Characteristics,” Clinical Neurophysiology 114, no. 9 (2003): 1580–1593. 10.1016/s1388-2457(03)00093-2 12948787

[33] S. D. Muthukumaraswamy , “High‐Frequency Brain Activity and Muscle Artifacts in MEG/EEG: A Review and Recommendations,” Frontiers in Human Neuroscience 7 (2013): 138. 10.3389/fnhum.2013.00138 PMC3625857 23596409

[34] B. B. Winter and J. G. Webster , “Driven‐Right‐Leg Circuit Design,” IEEE Transactions on Biomedical Engineering 30, no. 1 (1983): 62–66. 10.1109/tbme.1983.325168 6826188

[35] K. L. Snyder , J. E. Kline , H. J. Huang , and D. P. Ferris , “Independent Component Analysis of Gait‐Related Movement Artifact Recorded Using EEG Electrodes During Treadmill Walking,” Frontiers in Human Neuroscience 9 (2015): 639. 10.3389/fnhum.2015.00639 PMC4664645 26648858

[36] W. De Clercq , A. Vergult , B. Vanrumste , W. Van Paesschen , and S. Van Huffel , “Canonical Correlation Analysis Applied to Remove Muscle Artifacts From the Electroencephalogram,” IEEE Transactions on Biomedical Engineering 53, no. 12 (2006): 2583–2587. 10.1109/TBME.2006.879459 17153216

[37] N. A. Badcock , K. A. Preece , B. de Wit , et al., “Validation of the Emotiv EPOC EEG System for Research‐Quality Auditory Event‐Related Potentials,” PeerJ 1 (2013): e38. 10.7717/peerj.907 PMC4411518 25922794

